# Frailty and Sleep Disorder in Chronic Liver Diseases

**DOI:** 10.3390/life10080137

**Published:** 2020-08-05

**Authors:** Hiroki Nishikawa, Kazunori Yoh, Hirayuki Enomoto, Yoshinori Iwata, Takashi Nishimura, Shuhei Nishiguchi, Hiroko Iijima

**Affiliations:** 1 Department of Internal Medicine, Division of Gastroenterology and Hepatology, Hyogo College of Medicine, Nishinomiya 663-8501, Japan; mm2wintwin@ybb.ne.jp (K.Y.); enomoto@hyo-med.ac.jp (H.E.); yo-iwata@hyo-med.ac.jp (Y.I.); tk-nishimura@hyo-med.ac.jp (T.N.); hiroko-i@hyo-med.ac.jp (H.I.); 2 Center for Clinical Research and Education, Hyogo College of Medicine, Nishinomiya 663-8501, Japan; 3 Kano General Hospital, Osaka 531-0041, Japan; nishiguchi@heartfull.or.jp

**Keywords:** chronic liver disease, frailty, sleep disorder, PSQI-J

## Abstract

We aimed to investigate the association in frailty and sleep disorder as assessed by the Japanese version of Pittsburgh Sleep Quality Index (PSQI-J) in patients with chronic liver diseases (CLDs, *n* = 317, 141 males). Frailty was determined using the following five phenotypes: unintentional body weight loss, self-reported exhaustion, muscle weakness, slow walking speed, and low physical activity. Sleep disorder was defined as patients with PSQI-J score 6 or greater. Robust (phenotype, 0), prefrail (1 or 2 phenotypes) and frailty (3 phenotypes or greater) were observed in 101 (31.9%), 174 (54.9%) and 42 (13.2%), respectively. The median (interquartile range (IQR)) PSQI-J score was 4 (3, 7). Sleep disorder was found in 115 patients (36.3%). The median (IQR) PSQI-J scores in patients of the robust, prefrail, and frail groups were 3 (2, 5), 5 (3, 7), and 8 (4.75, 10.25), respectively (*p* < 0.0001 between any two groups and overall *p* < 0.0001). The ratios of sleep disorder in patients with robust, prefrail and frailty were 15.8% (16/101), 39.1% (68/174), and 73.8% (31/42), respectively (overall *p* < 0.0001). In conclusion, CLD patients with frailty can involve poorer sleep quality. As sleep disorder in CLDs is potentially remediable, future frailty-preventive strategies must take sleep complaints into account.

## 1. Introduction

Frailty is a condition in which physiological and psychological activities (motor function, cognitive function, etc.) decrease with aging, and living activities are decreased due to multiple underlying diseases [1,2,3]. Frailty indicates a status between being healthy and requiring nursing care [1,2,3]. Appropriate interventional strategies for frailty make it possible to maintain living activities [1,2,3]. The expansion of the disease entity of frailty to chronic liver diseases (CLDs) has been found these days [4,5,6,7,8,9]. In patients with poor liver function, frailty can occur independently of aging due to liver disease-specific protein-energy dysfunctions or other metabolic dysfunctions, which can be a point of focus among hepatologists [4,5,6,7,8,9]. 

Patients with CLD often describe sleep problems and the mechanisms of sleep disorders in such patients have not been fully investigated, however, they seem to be temporally linked to CLD itself [10,11,12,13,14,15,16,17,18,19,20,21,22]. Sleep disorder, which involves difficulties in falling asleep, intermittent awakening during the night and shortened total sleep duration, is a burden and negatively affects the quality of life, mood changes, and possibly fatigue, and all of them can result in adverse consequences [19,20]. Sleep disorders can negatively affect the immune system and are commonly linked to neurocognitive disorders in patients with CLD, independently of liver fibrosis severity [10,11,12,13]. The Pittsburgh Sleep Quality Index (PSQI) is a globally accepted method using questionnaires for the subjective assessment of sleep quality [23,24,25]. The association between sleep disorders in elderly people, such as night awakening and deterioration of sleep efficiency, and the onset of physical frailty has been reported [26,27,28]. Long sleep duration and excessive daytime sleepiness were associated with social frailty in community-dwelling elderly persons [29]. Sleep quality is reported to be associated with cognitive status, bodily pain, and vitality in elderly Japanese persons [30]. However, as far as we know, scarce data in the relevance of sleep disorder to frailty in patients with CLD are available. We sought to investigate the association in frailty and sleep disorder using the Japanese version of the PSQI (PSQI-J) in patients with CLD. 

## 2. Patients and Methods

### 2.1. Patients

This study was a cross-sectional and observational study with a retrospective nature. In our hospital, evaluation for nutritional status and physical activity and questionnaire for daily life have been routinely performed for patients who agreed. Thus, a total of 317 CLD patients with data for both frailty and PSQI-J score, who visited the Hyogo College of Medicine Hospital between July 2015 and March 2019, were analyzed. They included both inpatients and outpatients. Liver cirrhosis (LC) was determined as demonstrated previously [22]. Frailty was determined using the following five phenotypes: body weight (BW) loss without intention, exhaustion, muscle strength decline [grip strength (GS) < 26 kg in males and < 18 kg in females], slow walking speed (WS, < 1.0 m/s), and low physical activity (being unable to do light exercise). Frailty score ranged from 0 to 5 points using these five phenotypes. Patients with frailty phenotypes of 3 or more were defined as being frailty, while those with phenotypes of 1 or 2 were defined as being prefrail. Patients with no frailty phenotype were considered as being in the robust status [31,32]. GS and WS were tested as reported elsewhere [31,32,33]. Patients with large ascites or overt hepatic encephalopathy, with the high possibility of frailty, were not included due to their poor reliability in self-reported questionnaire.

### 2.2. PSQI-J Score

Sleep disorder was evaluated by the PSQI-J in this study [23,24,25]. In brief, 10 questions that form seven categories were included in the PSQI-J questionnaire [23,24,25]. Each category is rated on a scale of 0 to 3, and the sum of all categories is therefore up to 21 points. An elevated PSQI-J score indicates poorer sleep quality. Favorable predictability for sleep disorder was confirmed when the sum of each category was greater than 5 points [24]. Our patients were classified into normal sleeping habit (PSQI-J score: 0–5 points), mild sleep disorder (6–8 points), moderate sleep disorder (9–11 points), and severe sleep disorder (12 points or greater) [23,24,25]. 

### 2.3. Our Study

We studied the association in the frailty status and the PSQI-J score retrospectively. Approval for the study was obtained from the Ethical Committee of Hyogo College of Medicine Hospital. The protocol in the study rigorously followed all provisions of the 1975 Declaration of Helsinki. An opt-out method was used for the purpose of obtaining informed consent from the study subjects.

### 2.4. Statistics

JMP software (Version 14., SAS Institute Inc., Cary, NC, USA) was employed to analyze data statistically (*p* < 0.05 indicates significance). In the analysis of continuous parameters, Student’s *t*-test, Mann–Whitney *U*-test, analysis of variance or Kruskal–Wallis test was employed to evaluate the differences in the distribution of continuous parameters between groups. Continuous parameters were presented as medians and interquartile ranges (IQRs). Categorical parameters were presented as patient number and percentages. In the analysis of categorical parameters, the chi-squared test was employed to assess the group differences. Baseline variables with a *p* value <0.1 in our univariate analysis were subjected to the multivariate logistic regression analysis to select candidate variables.

## 3. Results

### 3.1. Patient Baseline Data

Table 1 summarized the baseline features (*n* = 317, 141 men and 176 women, median age = 65.0 years). Patients with albumin-bilirubin (ALBI) grade 1 accounted for 76.0% [34]. LC was identified in 115 patients (36.3%: Child Pugh A in 84 patients, B in 27 patients and C in 4 patients). Forty-three patients (13.6%) had slow WS. GS decrease was found in 24 patients (17.0%) in male and 48 patients (27.3%) in female. Exhaustion was reported in 161 patients (50.8%). BW loss was reported in 12 patients (3.8%). Low physical activity was reported in 80 patients (25.2%). Frailty score ranged from 0 to 5 (median of 1). Robust, prefrail and frail status were observed in 101 (31.9%), 174 (54.9%) and 42 (13.2%) patients, respectively. The median (IQR) PSQI-J score was 4 [3,7]. Sleep disorder (PSQI-J score of 6 points or greater) was observed in 115 patients (36.3%). There were 62 patients with mild sleep disorder, 33 with moderate sleep disorder, and 20 with severe sleep disorder. In 54 cases (17.0%: 24 LC patients (20.9%) and 30 non-LC patients (14.9%)), sleeping medications were used at least once a week. The median sleep duration in a day was 6.5 h. The proportion of sleep disorder in patients with non-LC, Child-Pugh A LC and Child-Pugh B or C LC were 67/202 (33.2%), 32/84 (38.1%) and 16/31 (51.6%), respectively (overall *p* = 0.1276). The proportion of frailty in patients with non-LC, Child-Pugh A LC and Child-Pugh B or C LC were 12/202 (5.9%), 17/84 (20.2%) and 13/31 (41.9%), respectively (overall *p* < 0.0001).

### 3.2. PSQI-J Scores for the Entire Cohort Stratified by Frailty Status

**Figure 1:**
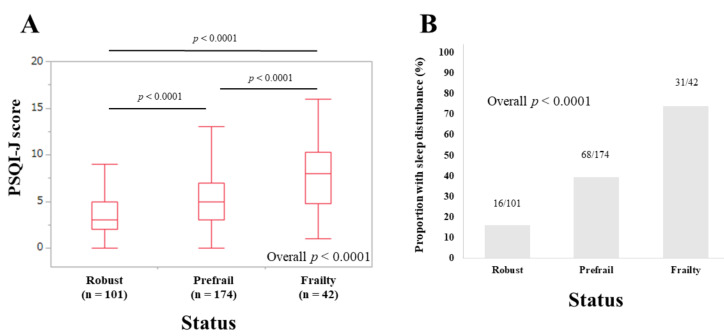
(**A**). PSQI-J scores in robust (*n* = 101), prefrail (*n* = 174) and frailty (*n* = 42) cases (*n* = 317). (**B**). The proportion of patients with sleep disorder in robust, prefrail and frail cases.

The median (IQR) PSQI-J scores in patients of the robust (*n* = 101), prefrail (*n* = 174) and frail (*n* = 42) groups were 3 (2, 5), 5 (3, 7), and 8 (4.75, 10.25), respectively (overall *p* < 0.0001) (Figure 1A). The corresponding ratios of patients with sleep disorder were 15.84% (16/101), 39.08% (68/174), and 73.81% (31/42), respectively (overall *p* < 0.0001) (Figure 1B).

### 3.3. Sleep Disorder according to the Five Phenotypes of Frailty

**Figure 2:**
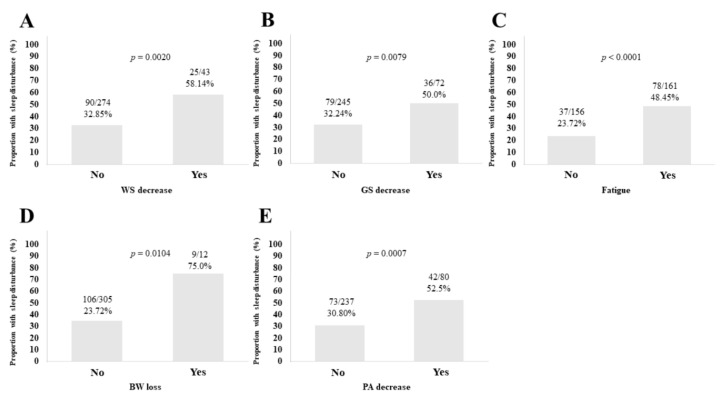
The proportion of sleep disorder according to five frailty phenotypes. (**A**) Walking speed (WS). (**B**) Grip strength (GS). (**C**) Fatigue. (**D**) Body weight (BW) loss. (**E**) Physical activity (PA).

WS decrease cohort had significantly higher ratio of sleep disorder than WS non-decrease cohort (*p* = 0.0020; Figure 2A). GS decrease cohort had significantly higher ratio of sleep disorder than GS non-decrease cohort (*p* = 0.0079; Figure 2B). Patients who reported fatigue had significantly higher ratio of sleep disorder than those who did not report fatigue (*p <* 0.0001; Figure 2C). Patients who reported BW loss had significantly higher ratio of sleep disorder than those who did not report BW loss (*p* = 0.0104; Figure 2D). PA decrease cohort had significantly higher ratio of sleep disorder than PA non-decrease cohort (*p* = 0.0007; Figure 2E). 

### 3.4. Subset Analysis 1: PSQI-J Scores for LC Patients Stratified by Frailty Status 

**Figure 3:**
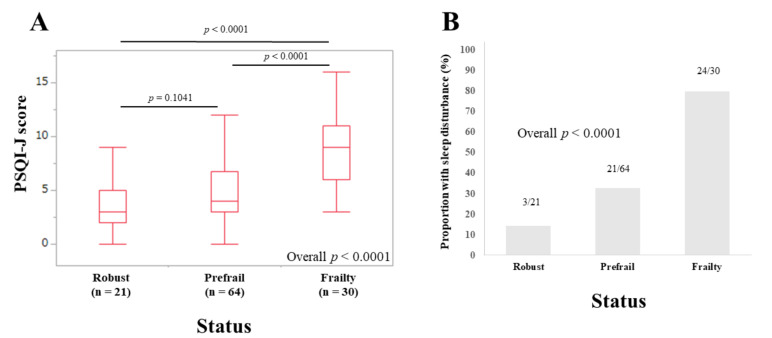
(**A**) PSQI-J scores in robust (*n* = 21), prefrail (*n* = 64) and frailty (*n* = 30) cases in patients with liver cirrhosis (*n* = 115). (**B**) The proportion of patients with sleep disorder in robust, prefrail, and frail cases in patients with liver cirrhosis.

The median (IQR) PSQI-J scores in LC patients with robust (*n* = 21), prefrail (*n* = 64) and frailty (*n* = 30) were 3 (2, 5), 4 (3, 6.75) and 9 (6, 11), respectively (overall *p <* 0.0001) (Figure 3A). The corresponding ratios of patients with sleep disorder were 14.29% (3/21), 32.81% (21/64), and 80.0% (24/30), respectively (overall *p* < 0.0001) (Figure 3B).

### 3.5. Subset Analysis 2: PSQI-J Scores for Non-LC Patients Stratified by Frailty Status

**Figure 4:**
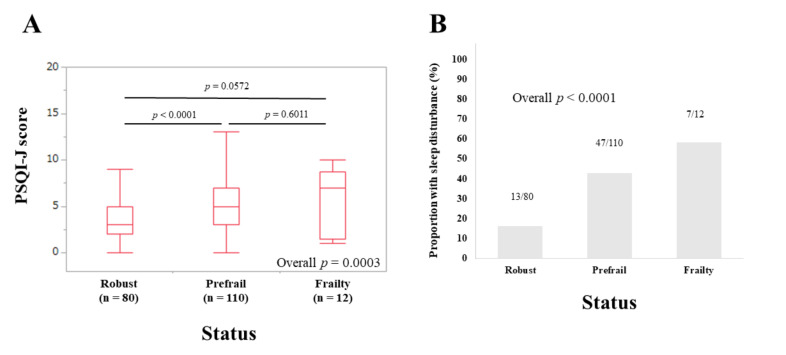
(**A**) PSQI-J scores in robust (*n* = 80), prefrail (*n* = 110) and frailty (*n* = 12) cases in patients with non-liver cirrhosis (*n* = 202). (**B**) The proportion of patients with sleep disorder in robust, prefrail, and frail cases in patients with non-liver cirrhosis.

The median (IQR) PSQI-J scores in non-LC patients with robust (*n* = 80), prefrail (*n* = 110) and frailty (*n* = 12) were 3 (2, 5), 5 (3, 7), and 7 (1.5, 8.75), respectively (overall *p* = 0.0003), (Figure 4A). The corresponding ratios of patients with sleep disorder were 16.25% (13/80), 42.73% (47/110), and 58.33% (7/12), respectively (overall *p <* 0.0001), (Figure 4B).

### 3.6. Subset Analysis 3: PSQI-J Scores for Elderly Patients (65 Years or Older) Stratified by Frailty Status

**Figure 5:**
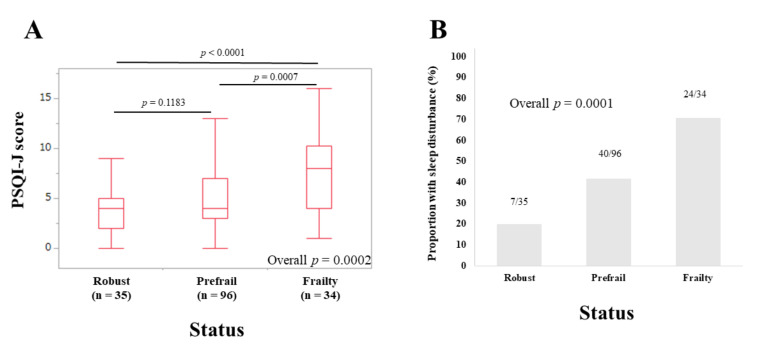
(**A**) PSQI-J scores in robust (*n* = 35), prefrail (*n* = 96) and frailty (*n* = 34) cases in patients aged 65 years or older (*n* = 165). (**B**) The proportion of patients with sleep disorder in robust, prefrail, and frail cases in patients aged 65 years or older.

The median (IQR) PSQI-J scores in elderly patients (65 years or older) with robust (*n* = 35), prefrail (*n* = 96) and frailty (*n* = 34) were 4 (2, 5), 4 (3, 7), and 8 (4, 10.25), respectively (overall *p* = 0.0002) (Figure 5A). The corresponding ratios of patients with sleep disorder were 20.0% (7/35), 41.67% (40/96), and 70.59% (24/34), respectively (overall *p =* 0.0001) (Figure 5B).

### 3.7. Subset Analysis 4: PSQI-J Scores for Non-Elderly Patients (<65 Years) Stratified by Frailty Status

**Figure 6:**
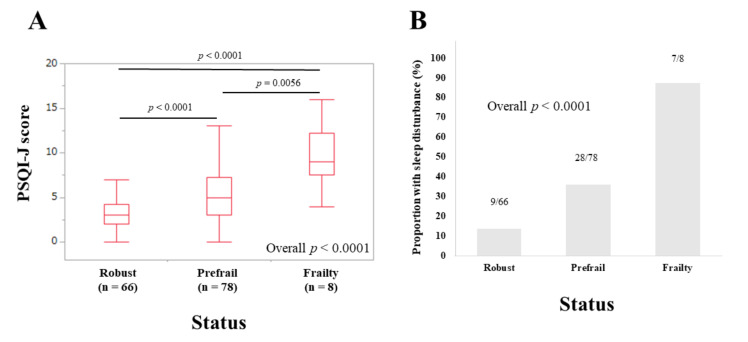
(**A**) PSQI-J scores in robust (*n* = 66), prefrail (*n* = 78), and frailty (*n* = 8) cases in patients less than 65 years (*n* = 152). (**B**) The proportion of patients with sleep disorder in robust, prefrail, and frail cases in patients less than 65 years.

The median (IQR) PSQI-J scores in non-elderly patients (<65 years) with robust (*n* = 66), prefrail (*n* = 78) and frailty (*n* = 8) were 3 (2, 4.25), 5 (3, 7.25), and 9 (7.5, 12.25), respectively (overall *p* < 0.0001) (Figure 6A). The corresponding ratios of patients with sleep disorder were 13.64% (9/66), 35.9% (28/78), and 87.5% (7/8), respectively (overall *p* < 0.0001) (Figure 6B).

### 3.8. Subset Analysis 5: PSQI-J Scores for Male Patients Stratified by Frailty Status

**Figure 7:**
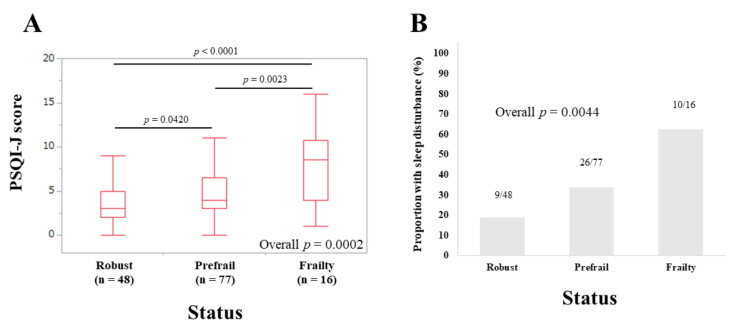
(**A**) PSQI-J scores in robust (*n* = 48), prefrail (*n* = 77), and frailty (*n* = 16) cases in male patients (*n* = 141). (**B**) The proportion of patients with sleep disorder in robust, prefrail, and frail cases in male patients.

The median (IQR) PSQI-J scores in male patients with robust (*n* = 48), prefrail (*n* = 77) and frailty (*n* = 16) were 3 (2, 5), 4 (3, 6.5), and 8.5 (4, 10.75), respectively (overall *p* = 0.0002) (Figure 7A). The corresponding ratios of patients with sleep disorder were 18.75% (9/48), 33.77% (26/77), and 62.5% (10/16), respectively (overall *p* = 0.0044) (Figure 7B).

### 3.9. Subset Analysis 6: PSQI-J Scores for Female Patients Stratified by Frailty Status

**Figure 8:**
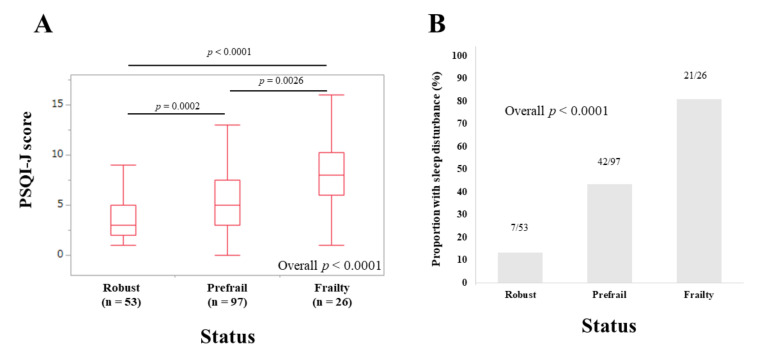
(**A**) PSQI-J scores in robust (*n* = 53), prefrail (*n* = 97) and frailty (*n* = 26) cases in female patients (*n* = 176). (**B**) The proportion of patients with sleep disorder in robust, prefrail, and frail cases in female patients.

The median (IQR) PSQI-J scores in female patients with robust (*n* = 53), prefrail (*n* = 97), and frailty (*n* = 26) were 3 (2, 5), 5 (3, 7.5), and 8 (6, 10.25), respectively (overall *p* < 0.0001) (Figure 8A). The corresponding ratios of patients with sleep disorder were 13.21% (7/53), 43.30% (42/97), and 80.77% (21/26), respectively (overall *p* < 0.0001) (Figure 8B).

### 3.10. Univariate and Multivariate Analyses of Factors Linked to Sleep Disorder for All Cases

As per the univariate analysis linked to sleep disorder (PSQI-J score 6 or greater) for all cases, age (*p* = 0.0111), and frailty score (*p* < 0.0001) were found to be significantly associated with sleep disorder, while ALBI score (*p* = 0.0825) and serum albumin (*p* = 0.0708) tended to be significant (Table 2). As per the multivariate analyses (because ALBI includes serum albumin, serum albumin was excluded in the multivariate analysis), only frailty score (*p* < 0.0001) was identified to be a significant factor (Table 2). Corresponding odds ratio (OR) and 95% confidence interval (CI) were shown in Table 2.

### 3.11. Univariate and Multivariate Analyses of Factors Linked to Sleep Disorder according to the LC Status

As per the univariate analysis linked to sleep disorder in LC patients, only frailty score (*p* < 0.0001) was found to be significantly associated with sleep disorder, while serum albumin tended to be significant (*p* = 0.0898) (Table 3). As per the multivariate analyses, only frailty score (*p* < 0.0001) was identified to be a significant factor (Table 3).

As per the univariate analysis linked to sleep disorder in non-LC patients, only frailty score (*p* = 0.0002) was found to be significantly associated with sleep disorder, while age (*p* = 0.0557) tended to be significant (Table 3). As per the multivariate analyses, only frailty score (*p* = 0.0023) was identified to be a significant factor (Table 3). Corresponding OR and 95% CI were shown in Table 3.

## 4. Discussion 

The investigation of sleep disorders and their relationship with frailty is an active topic and has been attracting increased attention. Prolonged sleep duration, insomnia, excessive napping, self-perception of reduced sleep quality, sleep onset latency, fragmented sleep, reduced sleep efficiency, sleep apnea, and daytime sleeping are reported to be independently linked to frail phenotypes [26,28,35,36]. However, to our knowledge, scarce data of the association in sleep disorder and frailty in patients with CLD are available. Patients with CLD are often suffering from long-standing disease burden, which may affect sleep quality [22]. In our data, the median sleep duration in a day was 6.5 h, which is almost identical to the average sleep duration of Japanese adults. Nevertheless, sleep disorder was identified in 115 patients (36.3%). These results actually reflect the adverse effect of CLD itself on sleep quality. 

In our results, PSQI-J scores and the proportion of patients with sleep disorder were well stratified among groups of robust, prefrail and frailty considering cases overall and all subgroups. In all five frailty phenotypes, the proportion of sleep disorder was also significantly stratified. In the multivariate analysis, frailty score was an independent predictor for the presence of sleep disorder regardless of the LC status. These results denoted that frailty in CLD can be closely associated with sleep disorder. Screening of sleep characteristics in patients with CLD may be relevant in order to identify patients at risk of frailty in the early phase. In our 42 frail patients, more than 70% had a sleep disorder, which appears to be a critical problem for the management of frail patients. The fact that about 40% of patients had a sleep disorder even in prefrail patients also seems to be an important issue. It is also worth reporting that none of our robust patients reported having very poor sleep quality in the PSQI-J questionnaire.

Moreno et al., demonstrated that sleep complaints were associated with increased risks of frailty in females, but not in males in elderly adults [37]. While in our data, both PSQI-J score and sleep disorder were well stratified among groups of robust, prefrail, and frail patients regardless of gender. Sex differences in the relationship between frailty and sleep disorder in patients with CLDs may be controversial. It is also of note that out of our eight frail patients under 65 years, seven had sleep disorder. Of these seven patients, six had LC. Consideration of frailty in patients with CLD should not be limited to elderly patients alone and disease-specific frailty condition should be fully considered even in younger patients with CLD [4,5,6,7,8]. Muscle protein synthetic ability can deteriorate even in younger patients with advanced CLD, primarily due to liver disease-specific protein energy malnutrition or other metabolic disorders [33]. Additionally, the psychological burden itself of suffering from LC may be involved in sleep disorders and is not dependent on age. 

In this study, frailty was determined by the Japanese version of cardiovascular health study criteria reported by Satake, et al. [31]. The five phenotypes (namely, WS, GS, fatigue, BW loss, and physical activity) mainly reflect physical frailty [31]. Our study results revealed that physical frailty in patients with CLDs can be linked to sleep disorder, but the relationship between sleep disorder and social or cognitive frailty in those with CLDs are unclear. On the other hand, improvement of physical activity has consistently been demonstrated to be associated with improvement of physical health, satisfaction with life, cognitive function, and psychological well-being, while conversely, a decline in physical activity can be associated with the occurrence of psychological disorders [38]. Exercise may ameliorate sleep disorders; however, whether exercise can improve sleep problems in frail patients with CLD needs further research [39,40].

Several limitations of our study, which must be acknowledged, were: (i) the current study was a single-center observational cross-sectional study with a retrospective nature; (ii) the study data originated from a Japanese CLD population, and additional data on others will be necessary; (iii) GS or WS can change depending on testing conditions; (iv) patients with large ascites or overt hepatic encephalopathy with the high possibility of frailty were not included in the study due to their poor reliability in self-reported questionnaire; (v) the causal relationship between frailty and sleep disorder was not known due to the cross-sectional study design of this study; (vi) covert or minimal hepatic encephalopathy which could influence on sleep quality was not assessed in this study [17]. Our data must thus be interpreted carefully. Nevertheless, our study data suggested that frailty in Japanese patients with CLD can be closely linked to sleep disorder. In conclusion, frail patients with CLD can have poorer sleep quality, and these trends may be observed even in prefrail patients. As sleep disorder in patients with CLDs is potentially remediable, future frailty-preventive strategies must take sleep complaints into account.

## Figures and Tables

**Table 1:** Baseline characteristics (*n* = 317).

Variable		All Cases (*n* = 317)
Age, years	Median (IQR)	65 (54, 72)
Gender	Male Female	141 (44.5%) 176 (55.5%)
Liver disease etiology	HCV HBV Other	169 (53.3%) 56 (17.7%) 92 (29.0%)
Presence of frailty	Yes No	42 (13.2%) 275 (86.8%)
Presence of LC	Yes No	115 (36.3%) 202 (63.7%)
Presence of ascites	Yes	13 (4.1%)
	No	304 (95.9%)
Presence of overt hepatic encephalopathy	Yes	0
	No	317 (100%)
Body mass index, kg/m^2^	Median (IQR)	22.9 (20.55, 25.8)
Walking speed, m/s	Median (IQR)	1.32 (1.14, 1.46)
Grip strength, kg, male	Median (IQR)	33.8 (28.3, 39.9)
Grip strength, kg, female	Median (IQR)	20.9 (17.6, 24.5)
Total bilirubin, mg/dL	Median (IQR)	0.8 (0.7, 1.1)
Serum albumin, g/dL	Median (IQR)	4.3 (4.0, 4.5)
ALBI score	Median (IQR)	−2.9 (−3.1, −2.6)
ALBI grade, *n* (%)	1 2 3	241 (76.0%) 69 (21.8%) 7 (2.2%)
Prothrombin time, %	Median (IQR)	91.1 (80.55, 98.6)
Platelet count, ×10^4^/mm^3^	Median (IQR)	17.7 (12.6, 21.85)
AST, IU/L	Median (IQR)	25 (19.5, 33.5)
ALT, IU/L	Median (IQR)	19 (14, 33)
Total cholesterol, mg/dL	Median (IQR)	182.5 (152, 214.75)
PSQI-J score	Median (IQR)	4 (3, 7)

IQR: interquartile range; HCV: Hepatitis C virus; HBV: hepatitis B virus; LC: liver cirrhosis; ALBI: albumin-bilirubin; AST: aspartate aminotransferase; ALT: alanine aminotransferase; PSQI-J: Japanese version of Pittsburgh Sleep Quality Index.

**Table 2:** Univariate and multivariate analyses of factors linked to sleep disorder for all cases.

Variables	Univariate *p*-Value	Multivariate (OR, Per One Unit)		
		OR	95%CI	*p*-Value
Age, Years	0.0111	1.048	0.988–1.030	0.4049
Gender, male/female	0.1597			
Presence of LC, yes/no	0.1452			
Etiology, HBV/HCV/others	0.7782			
Body mass index, kg/m^2^	0.3497			
Total bilirubin, mg/dL	0.5499			
Serum albumin, g/dL	0.0708			
ALBI score	0.0825	1.020	0.619–1.683	0.9372
Prothrombin time, %	0.8128			
Platelet count, ×10^4^/mm^3^	0.8237			
AST, IU/L	0.8981			
ALT, IU/L	0.5496			
Total cholesterol, mg/dL	0.8548			
Frailty score	<0.0001	1.939	1.505–2.498	<0.0001

LC: liver cirrhosis; HBV: hepatitis B virus; HCV: Hepatitis C virus; ALBI: albumin-bilirubin; AST: aspartate aminotransferase; ALT: alanine aminotransferase; OR: odds ratio; CI: confidence interval.

**Table 3:** Univariate and multivariate analyses of factors linked to sleep disorder according to the LC status.

	LC Patients				Non-LC Patients			
	Univariate *p*-Value	Multivariate (OR, Per One Unit)			Univariate *p*-Value	Multivariate (OR, Per One Unit)		
		OR	95%CI	*p*-Value		OR	95%CI	*p*-Value
Age, years	0.2562				0.0557	1.010	0.984-1.037	0.4549
Gender, male/female	0.1293				0.4502			
Etiology, HBV/HCV/others	0.2306				0.6555			
Body mass index, kg/m^2^	0.4048				0.6501			
Total bilirubin, mg/dL	0.9895				0.9537			
Serum albumin, g/dL	0.0898	0.954	0.454–2.006	0.9012	0.3658			
ALBI score	0.2494				0.3755			
Prothrombin time, %	0.4484				0.8401			
Platelet count, ×10^4^/mm^3^	0.1005				0.6948			
AST, IU/L	0.7656				0.5099			
ALT, IU/L	0.7553				0.3735			
Total cholesterol, mg/dL	0.5783				0.9321			
Frailty score	<0.0001	2.476	1.626–3.772	<0.0001	0.0002	1.711	1.212–2.416	0.0023

LC: liver cirrhosis; HBV: hepatitis B virus; HCV: Hepatitis C virus; ALBI: albumin-bilirubin; AST: aspartate aminotransferase; ALT: alanine aminotransferase; OR: odds ratio; CI: confidence interval.
